# Coagulation Status and Surgical Approach as Predictors of Postoperative Anemia in Patients Undergoing Thoracic Surgery: A Retrospective Study

**DOI:** 10.3389/fsurg.2021.744810

**Published:** 2021-09-21

**Authors:** Zhongping Lang, Yue Wu, Minwei Bao

**Affiliations:** ^1^Department of Laboratory Medicine, Shanghai Pulmonary Hospital Affiliated to Tongji University, Shanghai, China; ^2^Department of Thoracic Surgery, Shanghai Pulmonary Hospital Affiliated to Tongji University, Shanghai, China

**Keywords:** postoperative anemia, thoracic surgery, open thoracotomy, hemoglobin, prothrombin

## Abstract

**Objective:** Postoperative anemia is a common complication after a major surgery. Our study aims to identify factors that are associated with higher risk of developing postoperative anemia after thoracic surgery.

**Methods:** We conducted a retrospective study of 465 patients who underwent pulmonary surgery in 2017 in Shanghai Pulmonary Hospital, China. Of them, 191 patients underwent standard open thoracotomy (OT), and 274 patients underwent video-assisted thoracic surgery (VATS). A total of 350 patients were diagnosed with postoperative anemia, and 115 patients did not have anemia. Multiple logistic regression was used to compute odds ratios for predicting preoperative anemia.

**Results:** Postoperative anemia was associated with significantly lower weight (*p* < 0.001) and height (*p* = 0.022) of the patients, as well as higher prothrombin time (PT), and international normalized ratio (INR) (*p* = 0.012). Open thoracotomy resulted in a 1.2-fold increase in the incidence of postoperative anemia compared to VATS (*p* = 0.002). Multiple logistic regression analysis identified INR [OR (95% CI) 24.46 (2.05–292.27; *p* = 0.012] and surgical approach [OR (95% CI) 0.48 (0.31–0.74); *p* < 0.001] as predictors of postoperative anemia and postoperative drop in hemoglobin (Hb).

**Conclusion:** Postoperative coagulation status and surgical approach are statistically significant predictors of postoperative anemia in patients undergoing thoracic surgery. International normalized ratio and surgical approach are specifically associated with Hb drop immediately after the surgery.

## Introduction

Postoperative anemia is a common complication after a major surgery, and can be present in as high as 80–90% of surgical patients (1, 2). It may have deleterious effects on patients' health, and is associated with prolonged hospitalization, an increased rate of postoperative complications (especially infections), and overall reduced survival (3).

Numerous studies show that preoperative anemia, perioperative blood loss, and postoperative blunted erythropoiesis are the main contributing factors to postoperative anemia and increased transfusion need after major surgery (4). Other risk factors include low preoperative hemoglobin (Hb), female sex, age, perioperative coagulopathy, lower body surface area (5–8). Type of surgery performed also shows correlation with the rates of postoperative anemia after major operation (4). As a minimal invasive operation, video-assisted thoracic surgery (VATS) has been more and more widely used for the diagnosis and management of pathologies in the thoracic cavity due to improved wound infection rates, decreased chance of wound dehiscence, and reduced recovery times compared with open thoracotomy (OT) (9). However, VATS may potentially results in significant perioperative blood loss due to the procedure's proximity to large vessels (10, 11), that may potentially lead to postoperative anemia. Nevertheless, there is still not enough data on the possible predictors of anemia in patients after major surgeries. Identifying these risk factors will allow clinicians to specifically target patients who may require specific attention postoperatively or after discharge. The goal of this study is to identify risk factors that may predict postoperative anemia in patients, undergoing VATS or OT surgeries.

## Methods

The current retrospective study was conducted at Shanghai Pulmonary Hospital, China. Medical data of 465 patients that underwent pulmonary surgery in the year 2017 for benign or malignant conditions was analyzed. Pulmonary resection surgeries included 260 lobectomies, 45 pneumoectomies, 40 wedges, 110 segmentomies, and 10 lung transplants.

This study was approved by the ethics Tongji University Shanghai Pulmonary Hospital (No. K18-175). The data was routinely collected for all the patients, and included demographic, clinical and operational variables. Blood tests were done before and after surgery to measure Hb levels, prothrombin time (PT), international normalized ratio (INR), and activated partial thromboplastin time. Postoperative anemia was defined as a blood concentration of Hb <130g/L among men and <120g/L among women and was based on the WHO definition of anemia (12), and patients were stratified into two groups, patients with (*n* = 315) or without (*n* = 150) postoperative anemia. Postoperative Hb drop (g/L) was calculated based on the preoperative value.

Surgery was done under general anesthesia (GA) followed by intubation using a double lumen endotracheal tube in order to support lung ventilation. Patients, undergoing VATS (*n* = 274), were placed in the full lateral decubitus position and a thoracoscope was introduced through the 6^th^ or 7^th^ intercostal space on the mid-axillary line using a trocar. A 3–4 cm long utility incision was made through the 4^th^ or 5^th^ intercostal space on the anterior axillary line.

In cases (*n* = 191) where lesions invaded important blood vessels and structures, and minimally invasive surgery could not guarantee the surgical schedule, the surgeon opted to perform OT. The procedure included posterolateral thoracotomy accompanied by division of the latissimus dorsi and serratus anterior muscles.

Postoperative fluid management for all patient was 500–800 ml per day.

Postoperative anticoagulation therapy included enoxaparin sodium and heparin sodium injections and aspirin administration based on the instructions of the treating physician.

### Statistical Analysis

Categorical and binary variables were presented as counts and % of total and comparisons between cases with and cases without postoperative anemia were made using chi-square tests. Continuous variables were presented as mean and ± standard deviation (SD) and comparisons were made between cases with and cases without postoperative anemia using two-tailed independent samples *t*-tests. Demographic, clinical, and operative variables that were significant were included as independent variables in regression models for predicting postoperative. Independent variables were tested for multicollinearity using variance inflation factor. Multiple logistic regression was used to compute odds ratios for predicting preoperative anemia. Paired samples *t*-tests were performed within each independent variable to compare preoperative vs. postoperative concentrations of Hb. Relative change between preoperative and postoperative Hb was calculated as [(postoperative hemoglobin/preoperative hemoglobin – 1) × 100%]. For all tests, *p*-values < 0.05 were considered statistically significant.

## Results

Demographic characteristics of 465 patients that underwent pulmonary surgery and were included in the study are summarized in Table 1. Of them, 315 patients were diagnosed with postoperative anemia, and 150 had no postoperative anemia. The average age of study participants, mean (SD), was 60.14 (11.6), with no statistically significant difference between two groups.

**Table 1:** Comparison of demographic variables between cases with and cases without postoperative anemia.

		**With post-op**	**Without post-op**	
		**anemia**	**anemia**	
		**(*N* = 315)**	**(*N* = 150)**	***p*-values**
Gender	Male	212 (67.3)	112 (74.7)	0.106
	Female	103 (32.7)	38 (25.3)	
Age	years	60.4 (11.5)	59.6 (11.7)	0.517
Height	cm	165.0 (8.0)	167.4 (7.5)	0.003*
Weight	kg	61.1 (10.3)	67.3 (10.7)	<0.001*

All demographic variables compared between cases with and cases without postoperative anemia, with p < 0.05 considered statistically significant*. Binary variables are displayed as N (%) and compared using a chi-square test. Continuous variables are displayed as mean (±SD) and compared using a two-tailed independent samples t-test.

The average height and weight were significantly lower in patients who were diagnosed with postoperative anemia. The average weight of patients with anemia was 61.7 (10.3) vs. 67.3 (10.7) kg in the non-anemic group (*p* < 0.001), and the average height of postoperative and normal groups was 165.1 (8.0) and 166.8 (7.6) cm, respectively (*p* = 0.022).

Clinical variables of the study cohort are summarized in Table 2. Preoperative Hb was markedly lower in the postoperative anemia group [124.4 (17.1) vs. 142.9 (13.0), *p* < 0.001], which correlated with lower postoperative Hb [107.8 (14.9) vs. 136.5 (9.6) in the group of patients without anemia, *p* < 0.001]. Higher PT, and INR were slightly but statistically significantly associated with postoperative anemia (*p* = 0.012) (Table 2).

**Table 2:** Comparison of clinical variables between cases with and cases without postoperative anemia.

		**With post-op anemia**	**Without post-op anemia**	
		**(*N* = 315)**	**(*N* = 150)**	***p*-values**
Blood type	A	101 (32.1)	46 (30.7)	0.235
	B	76 (24.1)	48 (32.0)	
	AB	19 (6.0)	5 (3.3)	
	O	119 (37.8)	51 (34.0)	
Diabetes	Yes	25 (7.9)	10 (6.7)	0.628
	No	290 (92.1)	140 (93.3)	
Hypertension	Yes	50 (15.9)	27 (18.0)	0.564
	No	265 (84.1)	123 (82.0)	
Malignancy	Yes	245 (77.8)	127 (84.7)	0.083
	No	70 (22.2)	23 (15.3)	
Preoperative hemoglobin	g/L	124.4 (17.1)	142.9 (13.0)	<0.001
Postoperative hemoglobin	g/L	107.8 (14.9)	136.5 (9.6)	<0.001
Prothrombin time	s	11.3 (1.4)	11.0 (0.8)	0.012*
International normalized ratio		1.08 (0.12)	1.05 (0.07)	0.012*
Activated partial thromboplastin time	s	31.4 (3.6)	31.5 (3.7)	0.794
Length of admission	days	11.7 (6.5)	12.7 (13.7)	0.285

All clinical variables compared between cases with and cases without postoperative anemia, with p < 0.05 considered statistically significant*. Binary and categorical variables are displayed as N (%) and compared using a chi-square test. Continuous variables are displayed as mean (±SD) and compared using a two-tailed independent samples t-test.

Operative variables of the patients are summarized in Table 3. Surgical approach was significantly associated with postoperative anemia. Open thoracotomy was associated with a 1.2-fold increase in the incidence of postoperative anemia compared to VATS (*p* = 0.002), and with 1.45-fold increase in the units of transfused blood required (*p* < 0.001). Postoperative anticoagulant use was strongly associated with lower incidence of postoperative anemia. Sixty-eight percent of patients without anemia received postoperative anticoagulants, as compared to only 57.1% in the postoperative anemia group (*p* = 0.025).

**Table 3:** Comparison of operative variables between cases with and cases without postoperative anemia.

		**With post-op anemia**	**Without post-op anemia**	
		**(*N* = 315)**	**(*N* = 150)**	***p*-values**
Operation time	h	2.81 (1.12)	2.74 (1.05)	0.512
Total blood transfused	Units	7.1 (8.5)	6.0 (6.7)	0.148
Intraoperative blood loss	ml	451.8 (661.3)	492.6 (856.6)	0.574
Intraoperative blood transfusion	ml	290.2 (604.0)	398.0 (1264.9)	0.214
Preoperative anticoagulants	Yes	10 (3.2)	7 (4.7)	0.423
	No	305 (96.8)	143 (95.3)	
Postoperative anticoagulants	Yes	180 (57.1)	102 (68.0)	0.025*
	No	135 (42.9)	48 (32.0)	
Postoperative hemostatic use	Yes	166 (52.7)	71 (47.3)	0.279
	No	149 (47.3)	79 (52.7)	
Re-incision required to stop bleeding	Yes	11 (3.5)	6 (4.0)	0.785
	No	304 (96.5)	144 (96.0)	
		**VATS**	**Open**	
		**(** ***N*** **=** **274)**	**(** ***N*** **=** **191)**	***p*** **-values**
Postoperative anemia	Yes	170 (62.0)	145 (75.9)	0.002**
	No	104 (38.0)	46 (24.1)	
Total blood transfused	Units	5.7 (6.0)	8.3 (9.9)	0.001**

All operative variables compared between cases with and cases without postoperative anemia, with p < 0.05 considered statistically significant*. Binary variables are displayed as N (%) and compared using a chi-square test. Continuous variables are displayed as mean (±SD) and compared using a two-tailed independent samples t-test.

For VATS cases and open thoracotomy cases, all operative variables were compared with p < 0.05 considered statistically significant**. Continuous variables are displayed as mean (±SD) and compared using a two-tailed independent samples t-test.

We next performed regression analysis to identify factors that have a significant association with postoperative anemia (summarized in Table 4).

**Table 4:** Prediction of postoperative anemia from clinical and operative variables.

**Regression model**	**OR (95% CI)**	**Coefficient**	***p*-values**
Height	1.01 (0.97–1.04)	0.007	0.665
Weight	0.94 (0.92–0.97)	−0.058	<0.001*
INR	24.46 (2.05–292.27)	3.197	0.012*
Weight	0.95 (0.93–0.96)	−0.055	<0.001*
Post-op anticoag.	0.64 (0.42–0.97)	−0.454	0.036*
Weight	0.95 (0.93–0.97)	−0.055	<0.001*
Surgical approach	0.48 (0.31–0.74)	−0.736	<0.001*
Weight	0.94 (0.93–0.96)	−0.058	<0.001*
INR	17.48 (1.38–222.45)	2.861	0.027*
Post-op anticoag.	0.62 (0.40–0.95)	−0.485	0.029*
Surgical approach	0.48 (0.31–0.75)	−0.726	0.001*
Weight	0.94 (0.92–0.96)	−0.058	<0.001*

Surgical approach, post-operative anticoagulant use, and INR as independent variables in the prediction of postoperative anemia, defined as hemoglobin blood concentration <130 g/L among men and <120 g/L among women. Models have 1. All significant demographic variables as predictors; 2. 3., and 4. each significant clinical or operative variable as the independent variable adjusted for weight, and 5. all significant independent variables combined and adjusted for weight. Odds ratios are calculated using logistic regression, with p < 0.05* considered statistically significant.

When combined in a model, out of two demographic variables that were significantly associated with postoperative anemia, only weight remained significant and was used as an independent factor. Our analysis shows that INR [OR (95% CI) 24.46 (2.05–292.27; *p* = 0.012], and surgical approach [OR (95% CI) 0.48 (0.31–0.74); *p* < 0.001] remain statistically significant as predictors of postoperative anemia even when adjusted for weight (Table 4).

We next looked at the influence of the factors, significantly associated with postoperative anemia, on the postoperative changes in Hb levels (rather than just risk of low Hb, i.e., anemia). As summarized in Table 5, INR and surgical strategy were significantly associated with the observed drop in Hb (*p* < 0.001).

**Table 5:** Comparison of relative change in preoperative and postoperative hemoglobin among clinical and operative variables.

		**Preoperative hemoglobin**	**Postoperative hemoglobin**	**Relative change**	***p*-values**
Surgical	VATS	131.2 (17.9)	117.6 (19.4)	−10.4%	<0.001*
Approach	Open	129.2 (18.3)	116.2 (18.4)	−10.1%	<0.001*
International	High	127.6 (18.7)	115.3 (20.3)	−9.6%	<0.001*
normalized ratio	Low	133.1 (17.1)	118.7 (17.5)	−10.9%	<0.001*

Blood concentrations of hemoglobin (g/L) is listed as mean (±SD) for each of the three clinical and operative variables that were significantly associated with postoperative anemia. Relative change between preoperative and postoperative hemoglobin is compared within each variable indicating its potential impact on the reduction in hemoglobin. For each variable, preoperative and postoperative hemoglobin is compared for each of the binary categories using a two-tailed paired samples t-test, with p < 0.05^*^ considered statistically significant.

## Discussion

The data on the possible adverse effects of postoperative anemia on the recovery of surgical patients is limited. Generally, it is associated with worse morbidity- and mortality-related outcomes, and increased risks of readmission (4). Although multifactorial in origin, preoperative anemia, perioperative blood loss (surgical bleeding, coagulopathy, phlebotomies, etc.), and postoperative blunted erythropoiesis are the main contributing factors to postoperative anemia after major surgery.

The current study shows that postoperative coagulation profile and surgical approach are statistically significant as predictors of postoperative anemia in patients that underwent pulmonary surgery.

We found that lower weight and height correlated with higher incidence of postoperative anemia, with an average difference of 6.2 kg and 1.6 cm difference between patients, diagnosed with postoperative anemia, and a normal cohort. These results are in agreement with the previous studies that showed a significant association between anemia and BMI, and that smaller body surface area is a risk factor for the development of postoperative anemia and increased transfusion needs (13). When combined in a model where height and weight adjust for each other, only weight remained a significant risk factor for postoperative anemia.

Prior studies have reported that higher levels of anticoagulation may be considered as an independent risk factor of increased incidence of bleeding (14–17) in patients, treated with anticoagulants. Although INR of patients in both groups of our study was within a normal range, postoperative anemia was associated with slightly but significantly increased INR. Interestingly, we showed that postoperative anemia was more prevalent in the group that did not receive anticoagulant treatment after surgery. A possible explanation for these results may be that anticoagulants are less frequently prescribed for patients diagnosed with anemia to lower the risk of further bleeding.

Our study showed that OT is associated with a significantly higher risk of postoperative anemia than VATS. The current evidence in support of VATS over conventional OT is mainly based on observational non-randomized studies, national database analyses and institutional reports (18, 19). Studies in lung carcinoma patients showed that VATS was associated with a shorter duration of chest intubation, shorter hospitalization times, and overall improved survival (20). However, data is lacking on the correlation between surgery type and postoperative complications, such as bleeding and subsequent need for blood transfusion following bleeding. In our study, surgical approach had the most significant association with postoperative anemia and remained the strongest factor when combined in a regression model with INR and anticoagulant use [OR (95% CI) 0.48 (0.31–0.75); *p* = 0.001]. Open thoracotomy was also associated with 1.45-fold increase in the required units of transfused blood in surgical patients. Studies show that blood transfusion independently increases the risk of acute kidney injury in patients after cardiopulmonary surgery (21, 22). The increased requirement for blood transfusion in the OT cohort in our study may, therefore, suggest potentially increased postoperative morbidity.

Another variable, assessed in our study, was postoperative Hb drop immediately after surgery. Previous studies reported that the magnitude of postoperative downward Hb drift depends on the type of surgical procedure, as well as coagulation status of surgical patients (23, 24). Surgeries associated with greater intraoperative blood loss and postoperative bleeding are associated with a greater drop in Hb. In our study, coagulation status of patients (higher INR) had the greatest influence on reducing the postoperative drop in Hb (−9.6 vs. −10.9%). There was also a small, but still statistically significant association of VATS as a surgery method with a bigger postoperative Hb drop. Previous studies showed that the risk of injury to the pulmonary vessels leading to greater blood loss is higher in VATS compared to the standard OT, and is highly dependent on the skills of the operating surgeon (10, 25). We may speculate that this factor may contribute to the discrepancy between the overall lower incidence of postoperative anemia in VATS patients and larger initial postoperative Hb drop.

This study has some limitations. Since the study is retrospective, causality may be difficult to establish. Therefore, there is a risk that variables that were not assessed in the study may have affected the outcome of the analysis. Lung cancer staging for both groups was unavailable for analysis, and therefore, its potential impact on the type of surgery and postoperative anemia was not evaluated. There was no information on the type of chemotherapy, received by the patients prior to surgery, which may potentially affect the rates of preoperative and postoperative anemia. Additionally, OT in our study was performed on more difficult and complexed cases than VATS, which may introduce additional risk of biased outcome analysis. Randomized controlled studies are needed to further evaluate the effect of surgery type and coagulation status of patients on the risk of postoperative anemia.

## Conclusion

The current study shows that postoperative coagulation status of the patients and surgical approach are statistically significant predictors of postoperative anemia in patients undergoing pulmonary surgery. International normalized ratio and surgical approach correlate with Hb drop immediately after the surgery. These findings may help to develop more effective guidelines for efficient patient blood management that will reduce the need for blood transfusion, healthcare costs and morbidity and mortality.

## Data Availability Statement

The raw data supporting the conclusions of this article will be made available by the authors, without undue reservation.

## Author Contributions

ZL and YW conceived and designed the study. YW and MB were involved in literature search and data collection. ZL and YW analyzed the data. ZL and MB wrote the paper. YW reviewed and edited the manuscript. All authors read and approved the final manuscript.

## Conflict of Interest

The authors declare that the research was conducted in the absence of any commercial or financial relationships that could be construed as a potential conflict of interest.

## Publisher's Note

All claims expressed in this article are solely those of the authors and do not necessarily represent those of their affiliated organizations, or those of the publisher, the editors and the reviewers. Any product that may be evaluated in this article, or claim that may be made by its manufacturer, is not guaranteed or endorsed by the publisher.
